# Dynamic Changes in Quantitative MRI‐based Biomarkers and Its Association with Pathology of Alzheimer’s Disease – A Longitudinal Study

**DOI:** 10.1002/alz.089055

**Published:** 2025-01-09

**Authors:** Xiang Fan, Yuan Cai, Wanting Liu, Lei Zhao, Yishan Luo, Lin Shi, Vincent Chung Tong Mok

**Affiliations:** ^1^ Peking University Shenzhen Hospital, Shenzhen, Guangdong China; ^2^ Lau Tat‐chuen Research Centre of Brain Degenerative Diseases in Chinese, Gerald Choa Neuroscience Institute, Therese Pei Fong Chow Research Centre for Prevention of Dementia, The Chinese University of Hong Kong, Prince of Wales Hospital, Hong Kong SAR Hong Kong; ^3^ BrainNow Research Institute, Shenzhen China; ^4^ Department of Imaging and Interventional Radiology, The Chinese University of Hong Kong, Prince of Wales Hospital, Hong Kong SAR Hong Kong

## Abstract

**Background:**

Alzheimer’s disease (AD) resemblance atrophy index (AD‐RAI) is a novel MRI‐based machine‐learning derived biomarker for AD that is valid in the detection of early AD. We aimed to investigate the association between the serial change of AD‐RAI and cognitive progression overtime among cognitively unimpaired (CU) and mild cognitive impairment (MCI) subjects with and without A+T+ (i.e., Aβ+ and Tau+) and to compare it with other conventional MRI biomarkers (i.e., hippocampal volume (HV), hippocampal fraction (HF), brain parenchymal volume (BPV) and fraction (BPF)), which have been used in the past as surrogate markers for AD.

**Method:**

We selected participants from the ADNI databases and included those aged 55 to 90 years, diagnosed with CU and MCI at baseline with high‐quality volumetric MRI data and CSF at baseline, and at least once matched T1W scans and CSF during follow‐up. We define A+T+ based on the threshold value of Aβ_1‐42_ and ptau in CSF. A total of 168 participants were recruited with serial MRI scans and corresponding CSF in total. We assessed longitudinal changes of the MRI biomarkers (i.e., AD‐RAI, HV, HF, BPV, BPF) in correlation with changes in time and conversion status with and without A+T+. We used a linear mixed‐effects model (LMM) in SPSS 26.0 to explore the serial changes of the imaging biomarkers and interaction effects with and without A+T+ over time, and further explore the association between dynamic CSF Aβ_1–42_ and dynamic changes in the imaging biomarkers’ interaction effects over time.

**Result:**

AD‐RAI with A+T+ increased significantly faster than non‐A+T+ over time (P < 0.05), while HV and HF with A+T+ decreased significantly faster than non‐A+T+ over time (P < 0.05, respectively), which performed better than BPV/BPF. Serial AD‐RAI was significantly associated with changes in CSF Aβ_1–42_ over time, which outperforms other imaging biomarkers.

**Conclusion:**

Serial AD‐RAI outperforms other conventional biomarkers in correlating with AD pathologies. It may be used as a surrogate marker for monitoring disease progression or treatment response in AD.

